# Crystal structures of 2,5-di­azido-1,4-phenyl­ene di­acetate and 2,5-di­azido-1,4-phenyl­ene dibutyrate

**DOI:** 10.1107/S1600536814013762

**Published:** 2014-06-23

**Authors:** Florian Glöcklhofer, Johannes Fröhlich, Berthold Stöger, Matthias Weil

**Affiliations:** a Institute of Applied Synthetic Chemistry, Vienna University of Technology, Getreidemarkt 9/163, A-1060 Vienna, Austria; b Institute for Chemical Technologies and Analytics, Division of Structural Chemistry, Vienna University of Technology, Getreidemarkt 9/164-SC, A-1060 Vienna, Austria

**Keywords:** crystal structure, click chemistry, azides

## Abstract

The asymmetric units of the title compounds, C_10_H_8_N_6_O_4_, (I), and C_14_H_16_N_6_O_4_, (II), each contain half of the respective mol­ecule which is completed by inversion symmetry. The two molecules differ in the ester moiety (acetate *versus* butyrate) and the crystal symmetry is different, *i.e.* triclinic for (I) and monoclinic for (II). The di­azido­phenyl­ene moieties are essentially planar [maximum deviation of 0.0216 (7) Å for (I) and 0.0330 (14) Å for (II)], and the ester functionalities are almost perpendicular to these planes, making dihedral angles of 79.93 (3)° for (I) and 79.42 (6)° for (II). In the crystals of both (I) and (II), there are no significant inter­molecular inter­actions present.

## Abstract 2

2,5-Di­azido-1,4-phenyl­ene di­acetate and dibutyrate are the first structurally characterized representatives with a *trans*-di­azido­phenyl­ene entity. Both mol­ecules possess inversion symmetry; however, the compounds crystallize in different crystal systems (triclinic *versus* monoclinic).

## Chemical context   

In recent years, copper(I)-catalysed cyclo­addition of organic azides and alkynes towards 1,4-disubstituted triazoles attained immense inter­est in various fields of organic chemistry and became famous as the ‘cream of the crop’ of click chemistry (Moses & Moorhouse, 2007 ▸). In materials chemistry, this kind of reaction is often applied for the synthesis of functional polymers (Qin *et al.*, 2010 ▸).
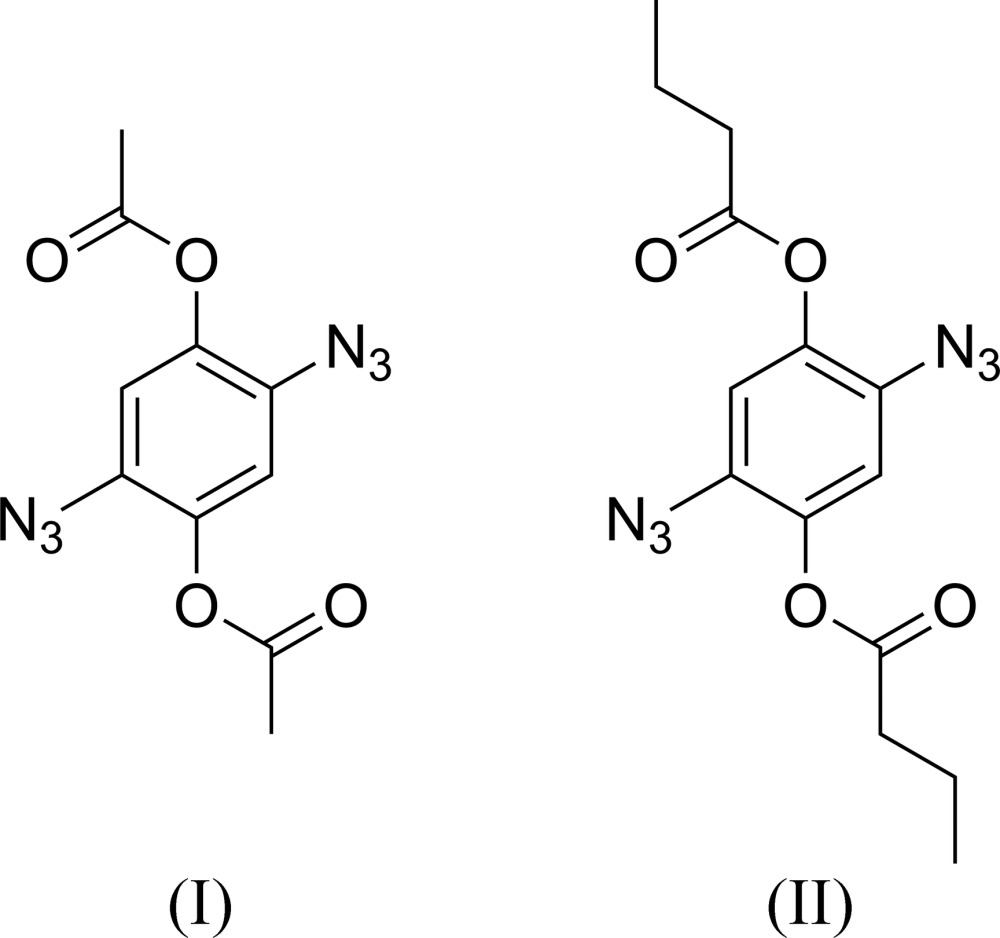


The title compounds, (I) and (II), were synthesized to investigate their applicability in such polymerizations, *viz. AA–BB* polymerizations with dialkynes. The synthetic accessibility of the two compounds from inexpensive starting materials is remarkable, making them suitable for large scale preparation. However, their electron-deficient character represents a challenge to the polymerization parameters. The crystal structures of (I) and (II) are reported herein.

## Structural commentary   

**Figure 1:**
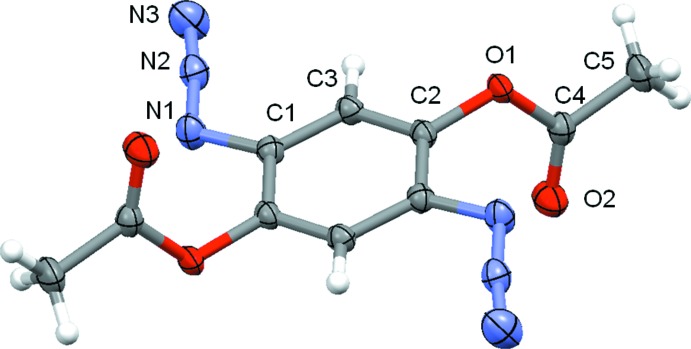
The mol­ecular structure of compound (I), showing the atom-labelling scheme. Displacement ellipsoids are drawn at the 80% probability level. Unlabelled atoms are generated by the symmetry code (−*x* + 1, −*y*, −*z*).

The mol­ecular structures of (I) and (II) are displayed in Figs. 1 ▸ and 2 ▸, respectively. Both mol­ecules possess inversion symmetry. Although the two mol­ecules differ only in the ester moiety (acetate *versus* butyrate), the crystal symmetry is different, *i.e.* triclinic for (I), with *Z* = 1, and monoclinic for (II), with *Z* = 2. The di­azido­phenyl­ene moieties do not differ significantly from planarity, with a maximum deviation of 0.0216 (7) Å in (I) and 0.0330 (14) Å in (II), for the unsubstituted atom C3 in both cases. The azide groups, both in *trans* positions to each other, deviate slightly from a linear arrangement, with an N—N—N angle of 173.01 (9)° for (I) and 172.59 (16)° for (II). The mean planes of the acetate [C—C(=O)—O)] and butyrate [C—C—C—C(=O)—O] groups are almost normal to the mean planes of the di­azido­phenyl­ene moieties, with a dihedral angle of 79.93 (3)° for (I) and 79.42 (6)° for (II).

## Supra­molecular features   

**Figure 3:**
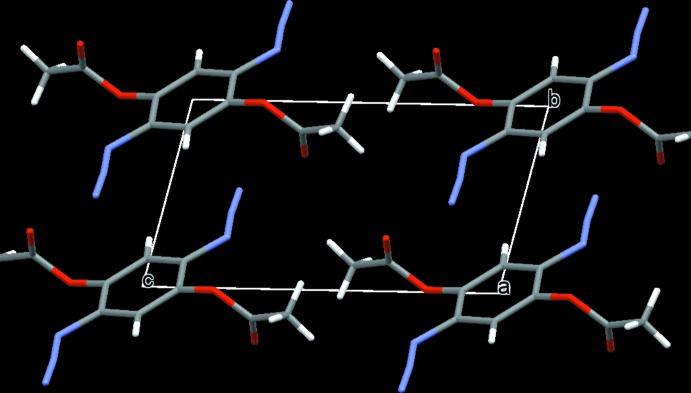
A view along [100] of the crystal packing of compound (I). Colour code: O red, C grey, N light-blue and H white.

There are no notable features in terms of π–π stacking inter­actions or hydrogen bonding in either structure. The crystal packing of (I) and (II) seems to be dominated mainly by van der Waals forces (Figs. 3 ▸ and 4 ▸, respectively).

## Database survey   

In the Cambridge Structural Database (Version 5.35, last update February 2014; Allen, 2002 ▸) no structures of compounds containing a *trans*-di­azido­phenyl­ene entity are listed, making the two examples presented herein the only ones reported so far.

## Synthesis and crystallization   

**Figure 5:**
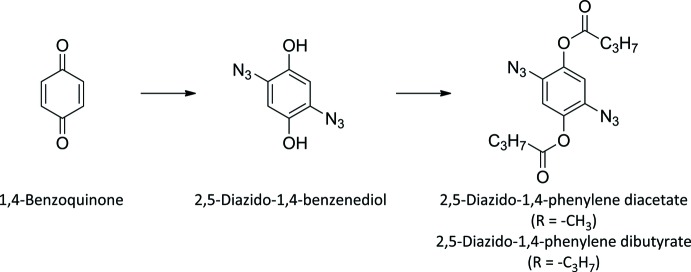
Reaction scheme for the synthesis of the title compounds.

Both target compounds were synthesized following a two-step protocol (Fig. 5 ▸), previously published for 2,5-di­azido-1,4-phenyl­ene di­acetate by Moore *et al.* (1969 ▸). In view of the light sensitivity of the inter­mediate compound 2,5-di­azido­benzene-1,4-diol, all reactions were carried out under light protection.

Preparation of 2,5-di­azido­benzene-1,4-diol: 1,4-benzo­quinone (10.81 g, 100.0 mmol, 1.0 equivalent) was dissolved in glacial acetic acid (100 ml, 1.0 *M*) and cooled to 288 K using an ice-water bath. NaN_3_ (14.3 g, 220 mol, 2.2 equivalents) was dissolved in water (44 ml, 5.0 *M*) and added to the cooled and stirred solution of 1,4-benzo­quinone in one portion. Stirring was stopped after 15 min and the flask was sealed and stored at 278 K overnight for crystallization. Vacuum filtration afforded a light-yellow solid, which was washed three times with water and dried *in vacuo* overnight to afford 2,5-di­azido­benzene-1,4-diol (yield: 6.60 g, 34.4 mmol, 69%). 1,4-Benzo­quinone serves as starting material and as oxidation reagent in this reaction, resulting in a theoretical molar yield of only half of the applied starting material (50 mmol).

Preparation of 2,5-di­azido-1,4-phenyl­ene di­acetate, (I): 2,5-di­azido­benzene-1,4-diol (1.92 g, 10.0 mmol) was added to preheated (313 K) acetic anhydride (100 ml, 0.1 *M*) in one portion and the reaction stirred until complete dissolution of the starting material. The reaction mixture was then allowed to cool to room temperature and stored overnight to allow 2,5-di­azido-1,4-phenyl­ene di­acetate to crystallize. Vacuum filtration afforded light-orange crystals of compound (I), which were washed with water three time (yield: 1.73 g, 6.26 mmol, 63%). ^1^H NMR (CDCl_3_, 200 MHz): δ 6.89 (*s*, 2H), 2.33 (*s*, 6H); ^13^C NMR (CDCl_3_, 50 MHz): δ 168.3 (*s*), 140.0 (*s*), 129.3 (*s*), 115.3 (*d*), 20.4 (*q*).

Preparation of 2,5-di­azido-1,4-phenyl­ene dibutyrate, (II): 2,5-di­azido­benzene-1,4-diol (1.34 g, 7.0 mmol) was added to preheated (333 K) butyric anhydride (20 ml, 0.35 *M*) in one portion and the resulting suspension stirred for 45 min at this temperature. The reaction mixture was then allowed to cool to room temperature and stored for 5 days to allow 2,5-di­azido-1,4-phenyl­ene dibutyrate to crystallize. Vacuum filtration afforded yellow crystals of compound (II), which were washed with water three times and with ethanol twice (yield: 814 mg, 2.45 mmol, 35%). ^1^H NMR (CDCl_3_, 200 MHz): δ 6.88 (*s*, 2H), 2.57 (*t*, *J* = 7.4 Hz, 4H), 1.80 (*sext*, *J* = 7.4 Hz, 4H), 1.05 (*t*, *J* = 7.4 Hz, 6H); ^13^C NMR (CDCl_3_, 50 MHz): δ 171.1 (*s*), 140.0 (*s*), 129.3 (*s*), 115.3 (*d*), 35.6 (*t*), 18.3 (*t*), 13.6 (*q*).

## Refinement   

For both structures, (I) and (II), the H atoms were included in calculated positions and treated as riding atoms, with C—H = 0.96 Å and *U*_iso_(H) = 1.2*U*_eq_(C). ▸

## Supplementary Material

Crystal structure: contains datablock(s) general, I, II. DOI: 10.1107/S1600536814013762/su0008sup1.cif

Structure factors: contains datablock(s) I. DOI: 10.1107/S1600536814013762/su0008Isup2.hkl

Structure factors: contains datablock(s) II. DOI: 10.1107/S1600536814013762/su0008IIsup3.hkl

Supporting information file. DOI: 10.1107/S1600536814013762/su0008Isup4.cml

Supporting information file. DOI: 10.1107/S1600536814013762/su0008IIsup5.cml

CCDC references: 1008063, 1008064

Additional supporting information:  crystallographic information; 3D view; checkCIF report

## Figures and Tables

**Figure 2:**
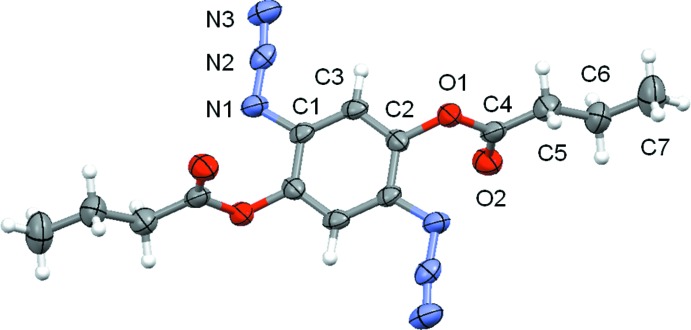
The mol­ecular structure of compound (II), showing the atom-labelling scheme. Displacement ellipsoids are drawn at the 80% probability level. Unlabelled atoms are generated by the symmetry code (−*x* + 1, −*y*, −*z*).

**Figure 4:**
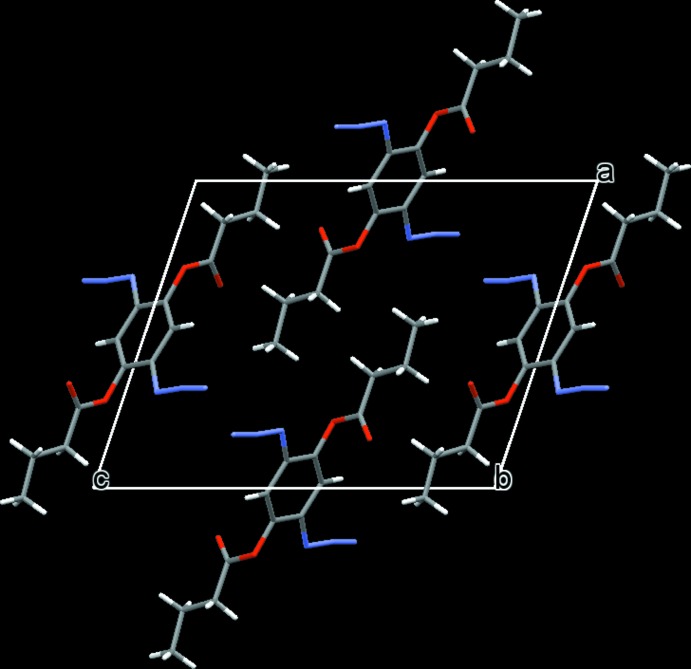
A view along [010] of the crystal packing of compound (II). Colour code: O red, C grey, N light-blue and H white.

**Table 1:** Experimental details

	(I)	(II)
Crystal data		
Chemical formula	C_10_H_8_N_6_O_4_	C_14_H_16_N_6_O_4_
*M*_r_	276.2	332.3
Crystal system, space group	Triclinic, *P* 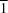	Monoclinic, *P*2_1_/*n*
Temperature (K)	100	100
*a*, *b*, *c* (Å)	5.4293 (6), 5.5678 (6), 10.4945 (12)	11.5875 (19), 5.1485 (8), 14.327 (2)
α, β, γ (°)	101.508 (3), 104.544 (3), 97.057 (3)	90, 108.496 (5), 90
*V* (Å^3^)	295.86 (6)	810.6 (2)
*Z*	1	2
Radiation type	Mo *K*α	Mo *K*α
μ (mm^−1^)	0.12	0.10
Crystal size (mm)	0.65 × 0.55 × 0.25	0.65 × 0.25 × 0.08

Data collection		
Diffractometer	Bruker Kappa APEXII CCD	Bruker Kappa APEXII CCD
Absorption correction	Multi-scan (*SADABS*; Bruker, 2013 ▸)	Multi-scan (*SADABS*; Bruker, 2013 ▸)
*T*_min_, *T*_max_	0.92, 0.97	0.97, 0.99
No. of measured, independent and observed [*I* > 3σ(*I*)] reflections	15989, 2182, 1983	17268, 1781, 1211
*R*_int_	0.037	0.043
(sin θ/λ)_max_ (Å^−1^)	0.764	0.662

Refinement		
*R*[*F* ^2^ > 2σ(*F* ^2^)], *wR*(*F* ^2^), *S*	0.034, 0.056, 3.22	0.042, 0.048, 2.15
No. of reflections	2182	1781
No. of parameters	91	109
H-atom treatment	H-atom parameters constrained	H-atom parameters constrained
Δρ_max_, Δρ_min_ (e Å^−3^)	0.46, −0.23	0.26, −0.23

Computer programs: *APEX2* and *SAINT-Plus* (Bruker, 2013 ▸), *SUPERFLIP* (Palatinus & Chapuis, 2007 ▸), *JANA2006* (Petříček, *et al.*, 2014 ▸), *Mercury* (Macrae *et al.*, 2008 ▸) and *publCIF* (Westrip, 2010 ▸).

## supplementary crystallographic information

### (I) 2,5-Diazido-1,4-phenylene diacetate Crystal data

**Table:**

C_10_H_8_N_6_O_4_	*V* = 295.86 (6) Å^3^
*M**_r_* = 276.2	*Z* = 1
Triclinic, *P*1	*F*(000) = 142
Hall symbol: -P 1	*D*_x_ = 1.550 Mg m^−^^3^
*a* = 5.4293 (6) Å	Mo *K*α radiation, λ = 0.71073 Å
*b* = 5.5678 (6) Å	θ = 3.8–32.8°
*c* = 10.4945 (12) Å	µ = 0.12 mm^−^^1^
α = 101.508 (3)°	*T* = 100 K
β = 104.544 (3)°	Irregular, light-orange
γ = 97.057 (3)°	0.65 × 0.55 × 0.25 mm

### (I) 2,5-Diazido-1,4-phenylene diacetate Data collection

**Table:**

Bruker Kappa APEXII CCD diffractometer	2182 independent reflections
Radiation source: X-ray tube	1983 reflections with *I* > 3σ(*I*)
Graphite monochromator	*R*_int_ = 0.037
ω and φ scans	θ_max_ = 32.9°, θ_min_ = 2.1°
Absorption correction: multi-scan (*SADABS*; Bruker, 2013)	*h* = −8→8
*T*_min_ = 0.92, *T*_max_ = 0.97	*k* = −8→8
15989 measured reflections	*l* = −15→16

### (I) 2,5-Diazido-1,4-phenylene diacetate Refinement

**Table:**

Refinement on *F*	16 constraints
*R*[*F* > 3σ(*F*)] = 0.034	H-atom parameters constrained
*wR*(*F*) = 0.056	Weighting scheme based on measured s.u.'s *w* = 1/(σ^2^(*F*) + 0.0001*F*^2^)
*S* = 3.22	(Δ/σ)_max_ = 0.011
2182 reflections	Δρ_max_ = 0.46 e Å^−^^3^
91 parameters	Δρ_min_ = −0.23 e Å^−^^3^
0 restraints	

### (I) 2,5-Diazido-1,4-phenylene diacetate Fractional atomic coordinates and isotropic or equivalent isotropic displacement parameters (Å^2^)

**Table:**

	*x*	*y*	*z*	*U*_iso_*/*U*_eq_	
O1	0.25671 (10)	0.01146 (10)	0.20279 (5)	0.01189 (16)	
O2	0.59540 (11)	0.28134 (10)	0.35546 (6)	0.01772 (18)	
N1	0.36886 (13)	0.27117 (12)	−0.19926 (6)	0.0141 (2)	
N2	0.19623 (12)	0.39755 (11)	−0.19328 (6)	0.01377 (19)	
N3	0.04446 (14)	0.51922 (13)	−0.19896 (8)	0.0208 (2)	
C1	0.42743 (13)	0.13837 (12)	−0.09681 (7)	0.0106 (2)	
C2	0.38596 (13)	0.01109 (13)	0.10373 (7)	0.01038 (19)	
C3	0.31388 (13)	0.14839 (12)	0.00901 (7)	0.0108 (2)	
C4	0.38466 (14)	0.15794 (13)	0.33011 (7)	0.0119 (2)	
C5	0.22479 (16)	0.13357 (15)	0.42495 (7)	0.0174 (2)	
H1c3	0.185611	0.250721	0.016023	0.013*	
H1c5	0.054271	0.162226	0.38606	0.0208*	
H2c5	0.213484	−0.031171	0.440361	0.0208*	
H3c5	0.303627	0.253962	0.509525	0.0208*	

### (I) 2,5-Diazido-1,4-phenylene diacetate Atomic displacement parameters (Å^2^)

**Table:**

	*U*^11^	*U*^22^	*U*^33^	*U*^12^	*U*^13^	*U*^23^
O1	0.0113 (3)	0.0152 (2)	0.0096 (2)	0.00109 (19)	0.00522 (18)	0.00195 (18)
O2	0.0164 (3)	0.0197 (3)	0.0148 (3)	−0.0021 (2)	0.0057 (2)	0.0004 (2)
N1	0.0169 (3)	0.0155 (3)	0.0139 (3)	0.0069 (2)	0.0072 (2)	0.0064 (2)
N2	0.0161 (3)	0.0132 (3)	0.0137 (3)	0.0024 (2)	0.0056 (2)	0.0056 (2)
N3	0.0214 (4)	0.0201 (3)	0.0276 (4)	0.0089 (3)	0.0118 (3)	0.0119 (3)
C1	0.0113 (3)	0.0107 (3)	0.0096 (3)	0.0016 (2)	0.0032 (2)	0.0021 (2)
C2	0.0105 (3)	0.0116 (3)	0.0091 (3)	0.0012 (2)	0.0042 (2)	0.0011 (2)
C3	0.0106 (3)	0.0115 (3)	0.0107 (3)	0.0027 (2)	0.0040 (2)	0.0018 (2)
C4	0.0144 (3)	0.0124 (3)	0.0102 (3)	0.0039 (2)	0.0049 (2)	0.0028 (2)
C5	0.0186 (4)	0.0228 (4)	0.0129 (3)	0.0030 (3)	0.0091 (3)	0.0037 (3)

### (I) 2,5-Diazido-1,4-phenylene diacetate Geometric parameters (Å, º)

**Table:**

O1—C2	1.3924 (10)	C1—C3	1.3943 (11)
O1—C4	1.3758 (8)	C2—C3	1.3810 (11)
O2—C4	1.1971 (9)	C3—H1c3	0.96
N1—N2	1.2456 (10)	C4—C5	1.4904 (12)
N1—C1	1.4167 (10)	C5—H1c5	0.96
N2—N3	1.1269 (10)	C5—H2c5	0.96
C1—C2^i^	1.3944 (11)	C5—H3c5	0.96
			
C2—O1—C4	116.60 (5)	C2—C3—H1c3	120
N2—N1—C1	115.40 (7)	O1—C4—O2	122.32 (7)
N1—N2—N3	173.01 (9)	O1—C4—C5	110.16 (6)
N1—C1—C2^i^	116.58 (7)	O2—C4—C5	127.51 (6)
N1—C1—C3	124.83 (7)	C4—C5—H1c5	109.47
C2^i^—C1—C3	118.59 (7)	C4—C5—H2c5	109.47
O1—C2—C1^i^	119.80 (7)	C4—C5—H3c5	109.47
O1—C2—C3	118.66 (7)	H1c5—C5—H2c5	109.47
C1^i^—C2—C3	121.42 (7)	H1c5—C5—H3c5	109.47
C1—C3—C2	120.00 (7)	H2c5—C5—H3c5	109.47
C1—C3—H1c3	120		

Symmetry code: (i) −*x*+1, −*y*, −*z*.

### (II) 2,5-Diazido-1,4-phenylene dibutyrate Crystal data

**Table:**

C_14_H_16_N_6_O_4_	*F*(000) = 348
*M**_r_* = 332.3	*D*_x_ = 1.361 Mg m^−^^3^
Monoclinic, *P*2_1_/*n*	Mo *K*α radiation, λ = 0.71073 Å
Hall symbol: -P 2yn	Cell parameters from 7352 reflections
*a* = 11.5875 (19) Å	θ = 2.7–27.0°
*b* = 5.1485 (8) Å	µ = 0.10 mm^−^^1^
*c* = 14.327 (2) Å	*T* = 100 K
β = 108.496 (5)°	Rod, light-yellow
*V* = 810.6 (2) Å^3^	0.65 × 0.25 × 0.08 mm
*Z* = 2	

### (II) 2,5-Diazido-1,4-phenylene dibutyrate Data collection

**Table:**

Bruker Kappa APEXII CCD diffractometer	1781 independent reflections
Radiation source: X-ray tube	1211 reflections with *I* > 3σ(*I*)
Graphite monochromator	*R*_int_ = 0.043
ω and φ scans	θ_max_ = 28.1°, θ_min_ = 2.0°
Absorption correction: multi-scan (*SADABS*; Bruker, 2013)	*h* = −13→14
*T*_min_ = 0.97, *T*_max_ = 0.99	*k* = −6→6
17268 measured reflections	*l* = −17→18

### (II) 2,5-Diazido-1,4-phenylene dibutyrate Refinement

**Table:**

Refinement on *F*	32 constraints
*R*[*F* > 3σ(*F*)] = 0.042	H-atom parameters constrained
*wR*(*F*) = 0.048	Weighting scheme based on measured s.u.'s *w* = 1/(σ^2^(*F*) + 0.0001*F*^2^)
*S* = 2.15	(Δ/σ)_max_ = 0.005
1781 reflections	Δρ_max_ = 0.26 e Å^−^^3^
109 parameters	Δρ_min_ = −0.23 e Å^−^^3^
0 restraints	

### (II) 2,5-Diazido-1,4-phenylene dibutyrate Fractional atomic coordinates and isotropic or equivalent isotropic displacement parameters (Å^2^)

**Table:**

	*x*	*y*	*z*	*U*_iso_*/*U*_eq_	
O1	0.28390 (9)	0.01294 (17)	0.04347 (7)	0.0217 (4)	
O2	0.33949 (10)	−0.32997 (19)	0.14715 (7)	0.0269 (4)	
N1	0.68785 (12)	0.3626 (2)	0.07891 (8)	0.0224 (5)	
N2	0.67449 (11)	0.5255 (2)	0.13953 (9)	0.0225 (5)	
N3	0.67447 (13)	0.6825 (2)	0.19484 (9)	0.0296 (5)	
C1	0.58925 (14)	0.1863 (2)	0.04072 (10)	0.0177 (5)	
C2	0.39470 (14)	0.0013 (3)	0.02402 (10)	0.0181 (5)	
C3	0.48219 (14)	0.1877 (3)	0.06446 (10)	0.0190 (5)	
C4	0.26254 (15)	−0.1781 (3)	0.10334 (10)	0.0214 (6)	
C5	0.13432 (15)	−0.1622 (3)	0.10373 (11)	0.0265 (6)	
C6	0.10348 (15)	−0.3423 (3)	0.17548 (11)	0.0303 (6)	
C7	−0.02989 (17)	−0.3320 (4)	0.16734 (13)	0.0411 (7)	
H1c3	0.468982	0.31648	0.108459	0.0228*	
H1c5	0.080047	−0.194143	0.038568	0.0318*	
H2c5	0.116086	0.013496	0.116656	0.0318*	
H1c6	0.152049	−0.298391	0.241353	0.0364*	
H2c6	0.125093	−0.51698	0.164467	0.0364*	
H1c7	−0.045158	−0.450116	0.21394	0.0493*	
H2c7	−0.050726	−0.158801	0.180958	0.0493*	
H3c7	−0.078376	−0.380637	0.101973	0.0493*	

### (II) 2,5-Diazido-1,4-phenylene dibutyrate Atomic displacement parameters (Å^2^)

**Table:**

	*U*^11^	*U*^22^	*U*^33^	*U*^12^	*U*^13^	*U*^23^
O1	0.0263 (7)	0.0157 (5)	0.0273 (6)	0.0017 (5)	0.0143 (5)	0.0033 (4)
O2	0.0324 (7)	0.0217 (6)	0.0303 (6)	0.0055 (5)	0.0150 (5)	0.0053 (5)
N1	0.0290 (9)	0.0153 (6)	0.0251 (7)	−0.0013 (6)	0.0114 (6)	−0.0036 (6)
N2	0.0264 (9)	0.0159 (6)	0.0247 (7)	−0.0015 (6)	0.0072 (6)	0.0037 (6)
N3	0.0397 (10)	0.0189 (7)	0.0297 (7)	−0.0022 (6)	0.0101 (7)	−0.0052 (6)
C1	0.0231 (10)	0.0108 (7)	0.0194 (7)	−0.0002 (6)	0.0069 (7)	0.0020 (6)
C2	0.0218 (10)	0.0148 (7)	0.0208 (8)	0.0040 (7)	0.0109 (7)	0.0048 (6)
C3	0.0283 (10)	0.0112 (7)	0.0189 (8)	0.0023 (6)	0.0095 (7)	0.0016 (6)
C4	0.0309 (11)	0.0135 (7)	0.0225 (8)	−0.0025 (7)	0.0124 (7)	−0.0029 (6)
C5	0.0286 (11)	0.0212 (8)	0.0320 (9)	0.0005 (7)	0.0131 (7)	0.0019 (7)
C6	0.0338 (11)	0.0259 (9)	0.0356 (9)	−0.0051 (8)	0.0173 (8)	0.0004 (7)
C7	0.0376 (12)	0.0516 (12)	0.0386 (11)	−0.0118 (9)	0.0184 (9)	0.0030 (9)

### (II) 2,5-Diazido-1,4-phenylene dibutyrate Geometric parameters (Å, º)

**Table:**

O1—C2	1.399 (2)	C4—C5	1.490 (3)
O1—C4	1.3780 (19)	C5—C6	1.509 (2)
O2—C4	1.2023 (17)	C5—H1c5	0.96
N1—N2	1.2518 (18)	C5—H2c5	0.96
N1—C1	1.4257 (18)	C6—C7	1.513 (3)
N2—N3	1.1318 (18)	C6—H1c6	0.96
C1—C2^i^	1.392 (2)	C6—H2c6	0.96
C1—C3	1.387 (2)	C7—H1c7	0.96
C2—C3	1.3825 (19)	C7—H2c7	0.96
C3—H1c3	0.96	C7—H3c7	0.96
			
C2—O1—C4	116.81 (11)	C4—C5—H2c5	109.47
N2—N1—C1	115.62 (14)	C6—C5—H1c5	109.47
N1—N2—N3	172.59 (16)	C6—C5—H2c5	109.47
N1—C1—C2^i^	115.94 (15)	H1c5—C5—H2c5	103.47
N1—C1—C3	124.96 (13)	C5—C6—C7	112.50 (13)
C2^i^—C1—C3	119.09 (13)	C5—C6—H1c6	109.47
O1—C2—C1^i^	119.18 (12)	C5—C6—H2c6	109.47
O1—C2—C3	118.96 (13)	C7—C6—H1c6	109.47
C1^i^—C2—C3	121.71 (15)	C7—C6—H2c6	109.47
C1—C3—C2	119.20 (14)	H1c6—C6—H2c6	106.26
C1—C3—H1c3	120.4	C6—C7—H1c7	109.47
C2—C3—H1c3	120.4	C6—C7—H2c7	109.47
O1—C4—O2	122.63 (16)	C6—C7—H3c7	109.47
O1—C4—C5	109.84 (12)	H1c7—C7—H2c7	109.47
O2—C4—C5	127.53 (15)	H1c7—C7—H3c7	109.47
C4—C5—C6	114.88 (12)	H2c7—C7—H3c7	109.47
C4—C5—H1c5	109.47		

Symmetry code: (i) −*x*+1, −*y*, −*z*.
